# Coumarin analogue 3-methyl-7H-furo[3,2-g]chromen-7-one as a possible antiparkinsonian agent

**DOI:** 10.7705/biomedica.4299

**Published:** 2019-09-01

**Authors:** María del Pilar Olaya, Nadezdha Esperanza Vergel, José Luis López, María Dolores Viña, Mario Francisco Guerrero

**Affiliations:** 1 Departamento de Farmacia, Facultad de Ciencias, Universidad Nacional de Colombia, Bogotá, D.C., Colombia Universidad Nacional de Colombia Departamento de Farmacia Facultad de Ciencias Universidad Nacional de Colombia BogotáD.C Colombia; 2 Departamento de Química Farmacéutica, Facultad de Farmacia, Universidad de Salamanca, Salamanca, España Universidad de Salamanca Departamento de Química Farmacéutica Facultad de Farmacia Universidad de Salamanca Salamanca Spain; 3 Departamento de Farmacología, Centro de Investigación en Medicina Molecular y Enfermedades Crónicas, Universidad de Santiago de Compostela, Santiago de Compostela, España Universidad de Santiago de Compostela Departamento de Farmacología Centro de Investigación en Medicina Molecular y Enfermedades Crónicas Universidad de Santiago de Compostela Santiago de Compostela Spain

**Keywords:** Parkinson’s disease, monoamine oxidase, coumarins, models, animal, reserpine, levodopa, haloperidol, antioxidants, enfermedad de Parkinson, monoaminooxidasa, cumarinas, modelos animales, reserpina, levodopa, haloperidol, antioxidantes

## Abstract

**Introduction:**

Parkinson’s disease is the second most common neurodegenerative disease. Monoamine oxidase B inhibitors are used in the treatment of this disease concomitantly with levodopa or as monotherapy. Several substituted coumarins have shown activity as inhibitors of monoamine oxidase B.

**Objective:**

To evaluate the possible antiparkinsonian effects of the coumarin analogue FCS005 (3-methyl-7H-furo[3,2-g]chromen-7-one) in mouse models, as well as its inhibitory activity towards monoamine oxidases (MAO) and its antioxidant activity.

**Materials and methods:**

FCS005 was synthesized and the reversal of hypokinesia was evaluated in the reserpine and levodopa models. Moreover, in the haloperidol model, its anticataleptic effects were evaluated. Additionally, the monoamine oxidase inhibitory activity and antioxidant activity of FCS005 were evaluated using *in vitro* and *ex vivo* studies, respectively.

**Results:**

FCS005 (100 mg/kg) caused the reversal of hypokinesia in the reserpine and levodopa models. This furocoumarin also presented anti-cataleptic effects at the same dose. Besides, it showed selective inhibitory activity towards the MAO-B isoform and antioxidant activity.

**Conclusion:**

These results attribute interesting properties to the compound FCS005. It is important to continue research on this molecule considering that it could be a potential antiparkinsonian agent.

## Resumen

**Introducción.:**

El segundo trastorno neurodegenerativo más común es la enfermedad de Parkinson. Los inhibidores de la monoamino oxidasa B se emplean en el tratamiento de esta enfermedad en monoterapia o concomitantemente con levodopa. Varios compuestos cumarínicos han mostrado actividad como inhibidores de la monoamino oxidasa B.

**Objetivo.:**

Evaluar los posibles efectos antiparkinsonianos del análogo de la cumarina FCS005 (3-methyl-7*H*-furo [3,2-*g* ] chromen-7-one) en modelos de ratones, la actividad inhibitoria frente a las monoamino oxidasas (MAO) y la actividad antioxidante.

**Materiales y métodos.:**

Se sintetizó la furanocumarina FCS005 y, en los modelos de reserpina y levodopa, se evaluó si producía reversión de la hipocinesia; en el modelo de haloperidol se evaluaron sus efectos anticatalépticos. Además, se evaluó *in vitro* la actividad inhibidora de MAO y, *ex vivo,* la actividad antioxidante del compuesto FCS005.

**Resultados.:**

El compuesto FCS005 en dosis de 100 mg/kg produjo la remisión de la hipocinesia en los modelos de reserpina y de levodopa. Esta furanocumarina presentó efectos anticatalépticos con la misma dosis. Además, mostró tener actividad inhibitoria selectiva sobre la MAO B, así como efectos antioxidantes.

**Conclusión.:**

Los resultados evidenciaron propiedades interesantes del compuesto FCS005. Es importante continuar investigando esta molécula porque puede ser un potencial agente antiparkinsoniano.

Parkinson’s disease is the second most common neurodegenerative disease after Alzheimer’s. The incidence of the disease ranges from 10 to 50/100,000 person-years with a prevalence between 100 and 300/100,000 population ^1^. Due to the general aging of the population, the number of Parkinson’s disease patients is expected to double by 2030 ^1^^,^^2^. The los of dopamine-secreting neurons within the *substantia nigra* and the presence of Lewy bodies are the major pathological findings in Parkinson’s disease ^3^ while resting tremor, bradykinesia, muscle rigidity, and alterations in balance and walking are its main motor symptoms ^4^^,^^5^.

Currently, levodopa (L-dopa) is the most widely used treatment for the disease. This symptomatic therapy compensates for the decreased level of dopamine (DA). In certain instances (e.g., mild symptoms, tremor as the only or most prominent symptom, aged <60 years), other medications as monoamine oxidase type B inhibitors may be used initially to avoid levodopa-related motor complications ^3^. The MAO-B inhibitors such as selegiline and rasagiline are a valuable adjunct therapy to L- DOPA for Parkinson’s disease ^6^. These agents improve the response to L-dopa in the later stages of the disease and are useful in the treatment of disease symptoms in early stages ^7^.

Several coumarin compounds present activity in the central nervous system (CNS). Coumarin (1,2-benzopyrone) obtained from the species *Hygrophila tyttha* Leonard has shown potential effects as an anticonvulsant, anxiolytic, and sedative ^8^. One study indicated that 4-propyl-2H-benzo[h]-chromen-2-one could have antidepressant effects ^9^. Different substituted coumarins have been synthesized and have shown activity as MAO-B inhibitors ^10^^-^^15^ and other coumarin derivatives have presented neuroprotective effects ^16^^,^^17^, which is important considering that currently it is expected that therapeutic alternatives control Parkinson’s disease symptoms and offer an approach to slow or stop the progression of neurodegeneration ^18^. Several coumarins have shown antioxidant activity ^19^^,^^20^, which has an interesting neuroprotective property as oxidative stress is among the main risk factors underlying nigral degeneration. Dopaminergic neurons in this region of the CNS are particularly susceptible to oxidative stress ^21^.

In this study, the coumarin FCS005 (3-methyl-7H-furo[3,2-g]chromen-7-one) was synthesized and its possible anti-parkinsonian effects were evaluated.

## Materials and methods

### Coumarin analogue

**Figure 1:**
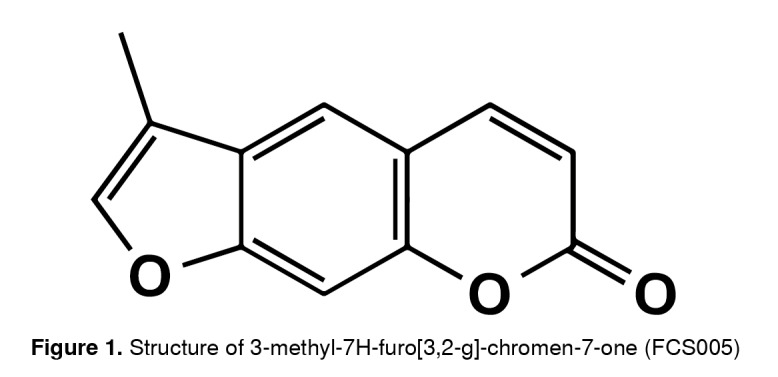
Structure of 3-methyl-7H-furo[3,2-g]-chromen-7-one (FCS005)

FCS005 is a furocoumarin obtained from a 7-hydroxylated coumarin following the experimental protocol previously described by Garazd, *et al.*^22^. It is an amorphous yellow powder with a melting point at 210-212°C and a molecular weight of 200 g/mol. The infrared and 1H/13C magnetic resonance spectra led to the elucidation of the structure of 3-methyl-7H-furo[3,2-g] chromen-7-one (figure 1) reported in the National Center for Biotechnology Information database ^23^.

Other reagents and drugs used in this investigation were selegiline, levodopa, carbidopa and reserpine, all supplied by Sigma-Aldrich. A MAO kit was obtained from Molecular Probes™.

### Animals

We used male ICR mice six to seven weeks old (25 - 30 g) provided by the bioterium of the *Departamento de Farmacia*, *Facultad de Ciencias*, at *Universidad Nacional de Colombia*. The animals were kept under standard laboratory conditions, 12-hour light-dark cycles, and at room temperature (22 ± 1°C) with food and water available *ad libitum.*

The effects of FCS005 (50, 100 and 200 mg/kg), selegiline (10 mg/ kg) or the vehicle on motor activity were evaluated at 1.5 and 23.5 h after administration in normal mice.

In the reserpine model and l-dopa/carbidopa assays, the locomotor activity was evaluated in the open-field test. The open field was a square arena divided into 16 squares with surrounding walls to prevent escape. Animals were placed individually in the middle of the open field and the specific parameter evaluated was the number of cadres traversed during 5 minutes. The open field was cleaned with a water-alcohol (10%) solution before placing the next animal to eliminate possible bias due to odors left by previous subjects.

### Evaluation of antiparkinsonian effects in the reserpine model

We followed the experimental protocol described by Tadaiesky, *et al.*^24^, with some modifications.

Reserpine 3 mg/kg was administrated intraperitoneally (i.p.) and 30 minutes later the mice were dosed with selegiline (10 mg/kg), FCS005 (50, 100 and 200 mg/kg) or vehicle by oral (p.o.) administration. The vehicle was a mixture of 15% propylene glycol, 15% glycerol, and enough distilled water to make up 100%. Locomotor activity was evaluated at 2 and 24 h after reserpine administration.

### Effect of L-dopa/carbidopa and MAO-B inhibitors in mice pretreated with reserpine

We evaluated the doses of FCS005 that showed significant differences in the reserpine model. The dose of L- dopa and carbidopa that did not cause the reversal of hypokinesia was determined in a dose-response relationship (data not shown). The mice were dosed with reserpine (3 mg/kg) i.p. 30 min before the p.o. administration of FCS005, selegiline or the vehicle. Levodopa plus carbidopa were administrated i.p. 30 min later. Locomotor activity was evaluated at 2 and 24 h after the administration of reserpine.

Anticataleptic effects and antioxidant activity

Haloperidol was administered to the animals to induce rigidity and akinesia (catalepsy) ^25^^,^^26^. Catalepsy was defined as the prolonged maintenance of both forepaws in an atypical position on a horizontal bar.

The animals received haloperidol (3 mg/kg) i.p.; FCS005 (100 mg/kg, p.o.), levodopa:carbidopa (400 mg/kg:40 mg/kg; p.o.) or vehicle (p.o) was administrated 30 min before. The reversal of catalepsy was evaluated for 2 min 60 min after the administration of the treatments.

Antioxidant activity was evaluated *ex vivo*. Mice were administered daily for 10 days with the treatments FCS005 (50 mg/kg, p.o.), levodopa:carbidopa (400 mg/kg:40 mg/kg, p.o.) or vehicle (p.o.) followed by haloperidol (1 mg/kg, i.p.) 30 min later. Then, the animals were killed by decapitation and the brains were removed, washed in 1% KCl, dissected on an ice-cold plate, and homogenized in 50 mM Tris-HCl buffer (pH 7.4). Homogenates were centrifuged (10,000 rpm for 10 min at 4°C). The final supernatant was stored at -20°C. The protein content of each sample was determined by the Bradford method.

The index of lipid peroxidation was evaluated following the protocol described by Hijova, *et al.*^27^. The brain homogenate (450 µL), 1 mL trichloroacetic acid (10%), and 50 µL of 50 mM phosphate buffer (pH 7.4) were centrifuged at 1850 xg for 10 min at 4°C; then, 1 mL of thiobarbituric acid (0.67%) was added to 1 mL of the supernatant. This mixture was heated to 92°C for 30 min and then cooled in an ice bath (4°C) before the absorbance was measured at 532 nm. The results were expressed as thiobarbituric acid reactive substances (TBARS) in mmol/mL/mg of tissue protein.

Quantification of protein carbonyl groups was determined by the technique of Levine, *et al.*^28^, following the protocol described by Baltacioglu, *et al.*^29^. Briefly, 250 µL of 10 mM 2,4-dinitrophenylhydrazine (DNPH) or 250 µL of 2 M HCl were added to the brain homogenate (50 µL) for the sample or the blank, respectively. The samples were left at room temperature in the dark for 1 h and vigorously stirred every 15 min. Then, 500 µL of trichloroacetic acid (20%) was added. The samples were kept in an ice bath for 15 min and then centrifuged at 11,000 rpm for 5 min. The pellet was washed three times with 1 mL of ethyl acetate/ethanol (1:1) solution. After each wash, the sample was centrifuged at 3,000 rpm for 7 min. Then, 250 µL of 6 M guanidine hydrochloride were added to the pellet to dissolve it and then it was incubated at 37°C for 10 min. The absorbance was determined at 360 nm. The content of carbonyl groups was calculated based on the molar extinction coefficient of DNPH (ε = 22 000 cm-1M-1) and expressed as nmol/mg protein ^29^.

### Assay of human monoamine oxidase (hMAO) inhibitory activity

The effects of FCS005 on hMAO isoform enzymatic activity were evaluated using a MAO kit (Molecular Probes™) using the experimental protocol previously described by Yáñez, *et al.*^30^. Briefly, adequate amounts of recombinant hMAO-A (1.1 mg protein; specific activity: 150 nmol p-tyramine oxidized to p-hydroxyphenylacetaldehyde/min/mg protein) or hMAO-B (7.5 mg protein; specific activity: 22 nmol *p*-tyramine transformed/min/mg protein) were added to 0.1 mL of sodium phosphate buffer (0.05 M, pH 7.4) to obtain the same reaction velocity and FCS005 in various concentrations. This mixture was incubated for 15 min at 37°C in a flat, black-bottomed 96-well microtest plate in a dark fluorimeter chamber. Then, the reaction was started by adding (final concentrations) 200 µM Amplex™ Red reagent, 1 U/mL horseradish peroxidase, and 1 mM *p*-tyramine. The production of H2 O _2_ and, consequently, of resorufin was quantified at 37°C in a multi-detection microplate fluorescence reader (FLX800TM, Bio-Tek Instruments, Inc., Winooski, VT, USA) based on the fluorescence generated (emission: 590 nm, excitation: 545 nm) over a 15-min period in which the fluorescence increased linearly.

The test drugs were added to solutions containing only the Amplex™ Red reagent in a sodium phosphate buffer to determinate the capacity of FCS005 or the reference inhibitors to modify the fluorescence generated in the reaction mixture due to non-enzymatic inhibition. In the control experiments, the test drugs were replaced with dilutions of the vehicles. In addition, the specific fluorescence emission was calculated after subtraction of the background activity, which was determined from wells containing all components except the hMAO isoforms that were replaced by a sodium phosphate buffer solution.

### Statistical analysis

Results were expressed as mean ± standard error of the mean (SEM) and assessed by a one-way analysis of variance (ANOVA) followed by the Tukey test to determine the treatments responsible for the significant differences. When the variance was not homogeneous or the data did not show normal distribution, the Kruskal-Wallis test was applied. The data on catalepsy were processed by a two-way analysis of variance (ANOVA) with the Bonferroni *post hoc* test. The graphical representation and statistical analysis were performed using GraphPad Prism (v. 5.03)

### Ethical aspects

All the experimental protocols were evaluated and approved by the ethics committee of the *Facultad de Ciencias*, *Universidad Nacional de Colombia* (Minutes No. 06, October 18, 2011).

## Results

### Mice not pretreated with reserpine

**Figure 2:**
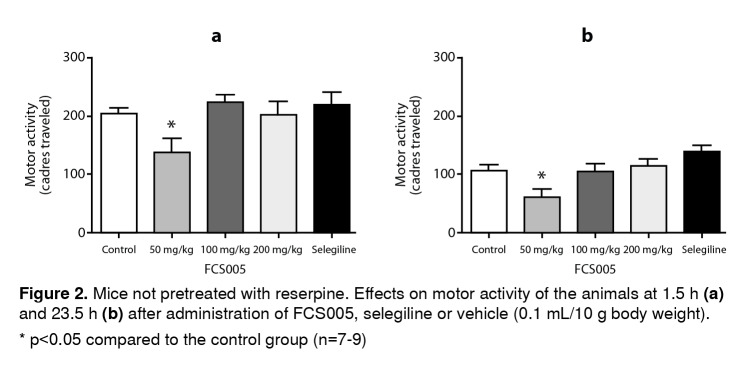
Mice not pretreated with reserpine. Effects on motor activity of the animals at 1.5 h **(a)** and 23.5 h **(b)** after administration of FCS005, selegiline or vehicle (0.1 mL/10 g body weight). * p<0.05 compared to the control group (n=7-9)

The compound FCS005 (50 mg/kg) caused a significant decrease in the locomotor activity of mice not pretreated with reserpine (figure 2) compared to the control group, both at 2 hours (p: 0.023) and at 24 hours (p: 0.003) post reserpine administration.

### Antiparkinsonian effects

**Figure 3:**
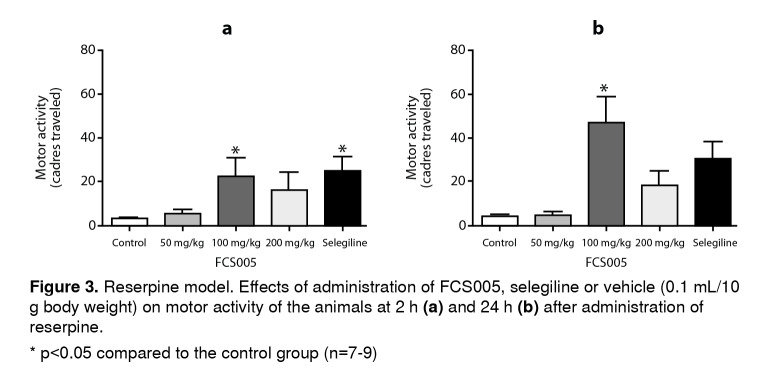
Reserpine model. Effects of administration of FCS005, selegiline or vehicle (0.1 mL/10 g body weight) on motor activity of the animals at 2 h **(a)** and 24 h **(b)** after administration of reserpine. * p<0.05 compared to the control group (n=7-9)

In the reserpine model, we observed that FCS005 (100 mg/kg) caused a reversal of hypokinesia produced by reserpine significantly increasing the number of cadres traversed compared to the control at 2 h (p: 0.007) and 24 h (p: 0.002) (figure 3).

### Effect of L-dopa/carbidopa and MAO-B inhibitors in mice pretreated with reserpine

**Figure 4:**
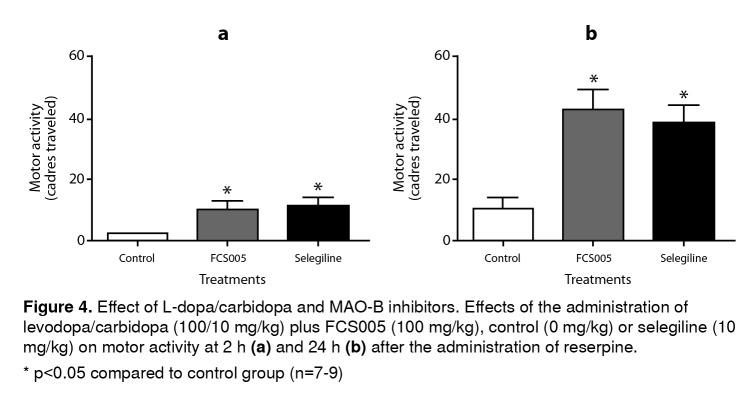
Effect of L-dopa/carbidopa and MAO-B inhibitors. Effects of the administration of levodopa/carbidopa (100/10 mg/kg) plus FCS005 (100 mg/kg), control (0 mg/kg) or selegiline (10 mg/kg) on motor activity at 2 h **(a)** and 24 h **(b)** after the administration of reserpine. * p<0.05 compared to control group (n=7-9)

We evaluated the effect of concomitant administration of MAO-B inhibitors and L-dopa at low doses. We used the dose 100:10 mg/kg of L-dopa:carbidopa given that in the dose-response relationship this dose did not lead to the reversal of hypokinesia (data not shown). We evaluated the dose of FCS005 (100 mg/kg) that showed the best response in the reserpine model. In this assay, FCS005 increased the number of cadres traversed showing a statistically significant difference compared to the control at 2 h (p: 0.038) and 24 h (p: 0.018) (figure 4).

### Anticataleptic effects

**Figure 5:**
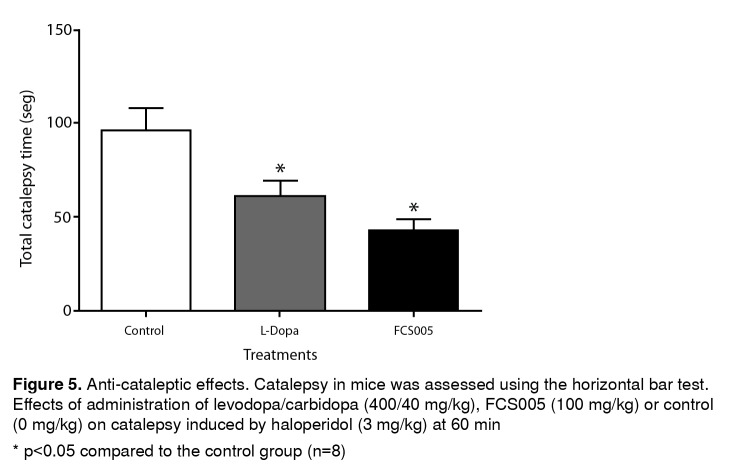
Anti-cataleptic effects. Catalepsy in mice was assessed using the horizontal bar test. Effects of administration of levodopa/carbidopa (400/40 mg/kg), FCS005 (100 mg/kg) or control (0 mg/kg) on catalepsy induced by haloperidol (3 mg/kg) at 60 min * p<0.05 compared to the control group (n=8)

The treatments showed no cataleptic effects at time zero. L-dopa/ carbidopa and FCS005 produced a significant decrease in the total catalepsy time using the horizontal bar test at 60 min (p: 0.001) (figure 5).

### Antioxidant activity

**Figure 6:**
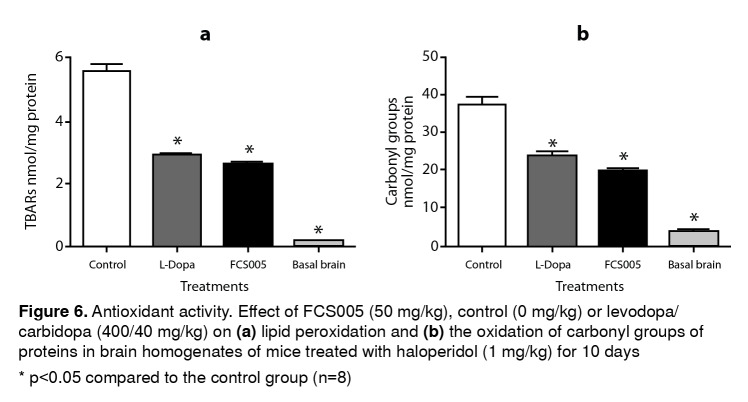
Antioxidant activity. Effect of FCS005 (50 mg/kg), control (0 mg/kg) or levodopa/ carbidopa (400/40 mg/kg) on **(a)** lipid peroxidation and **(b)** the oxidation of carbonyl groups of proteins in brain homogenates of mice treated with haloperidol (1 mg/kg) for 10 days * p<0.05 compared to the control group (n=8)

Protein carbonyl groups and lipid peroxidation levels were lower in brain homogenates of mice treated with FCS005 and L-dopa/carbidopa compared to the control animals. The animals that did not receive haloperidol (basal state) presented lower levels than those of the control and treatments groups (p<0.001) (figure 6).

### In vitro inhibition of the A and B isoforms of MAO

FCS005 presented selective inhibitory activity towards MAO-B. This coumarin analogue did not inhibit MAO-A isoform activity at the highest concentration tested (100 µM). The reference inhibitors and FCS005 did not interfere with the measurements because these drugs did not react directly with the Amplex™ Red reagent. The corresponding IC50 values and the selectivity index [IC50 (hMAO-A) ]/[IC50 (hMAO-B) ] are shown in table 1.

**Table 1:** *In vitro* inhibitory activities of reference compounds and FCS005 towards hMAO-A and hMAO-B isoformsa

Compounds	hMAO-A (IC_50_)	hMAO-B (IC_50_)	Selectivity index^b^
FCS005	*	41.63 ± 2.79 μM	> 2.4^c^
Clorgiline	4.46 ± 0.32 nM	61.35 ± 1.13 μM	0.000073
Selegiline	67.25 ± 1.02 μM	19.60 ± 0.86 nM	3.431
Iproniazide	6.56 ± 0.76 μM	7.54 ± 0.36 μM	0.87

a Each IC50 value is the mean ± S.E.M. from five experiments (n=5).

b hMAO-B selectivity ratios [IC50 (hMAO-A) ]/ [IC50 (hMAO-B) ] for inhibitory effects of FCS005 compound and reference inhibitors

c Values obtained under the assumption that the corresponding IC50 against hMAO-A is the highest concentration tested (100 µM)

* Inactive at 100 µM (highest concentration tested)

## Discussion

The antiparkinsonian activity of FCS005 was evaluated in mice using the reserpine model. The effect of reserpine on spontaneous locomotor activity is frequently used as a model of motor disturbances in Parkinson’s disease ^24^^,^^31^^,^^32^^,^^33^. Some drugs currently on the market were tested in this model suggesting it has predictive validity ^24^^,^^33^.

Hypokinesia occurs in mice because reserpine blocks the vesicular monoamine transporter and produces a profound and lasting decrease in catecholamines, a situation that results in the depletion of DA in all dopaminergic nerve terminals including the nigrostriatal pathway ^14^^,^^18^. FCS005 (100 mg/kg) caused the significant reversal of hypokinesia in the reserpine and levodopa models. This could be explained by the fact that the synthetic coumarin FCS005 showed selective inhibitory activity against hMAO-B *in vitro*. MAO inhibitors reduce the enzymatic degradation of dopamine by MAO, thus leading to an increase in monoamines and striatal dopaminergic activity and improving motor symptoms in Parkinson´s disease ^34^^,^^35^. Dopamine acts on postsynaptic D1 and D2 receptors to control movement ^36^. The selective inhibitory activity on MAO type B prolongs the activity of both endogenously and exogenously derived dopamine, which represents an option either as monotherapy in early Parkinson’s disease or as adjuvant therapy in patients treated with levodopa when they experience motor complications ^35^. Selegiline and rasagiline, selective inhibitors of MAO-B, are used in clinical practice to improve the response to L-dopa at later stages of the disease ^7^.

FCS005 at a dose of 50 mg/kg led to a significant decrease in motor activity in mice not treated with reserpine. However, this did not affect the evaluation in the models of reserpine and levodopa where the reversal of hypokinesia was evaluated. On the other hand, the decrease in motor activity at 24 h with all treatments could be attributed to habituation that occurs due to repeated exposure to the open field ^37^^,^^38^.

The coumarin analogue FCS005 showed anti-cataleptic effects in the haloperidol model. The animals presented catalepsy and muscular rigidity because haloperidol blocks nigrostriatal dopamine transmission ^39^^,^^40^. These symptoms may be analogous to the inability of Parkinson’s disease patients to initiate movements ^40^. FSC005 did not show any cataleptic effects.

Some studies have shown that chronic administration of haloperidol induces increased levels of lipid peroxidation and decreased levels of antioxidant enzymes and reduced glutathione ^41^^,^^42^. Besides, the results of Martins, *et al.*^43^ showed that TBARS increased in the striatum after the administration of haloperidol in repeated doses. In this study, an increase in the TBARS concentration was also observed, possibly because dopamine receptors were blocked by haloperidol leading to an increase in dopamine and, therefore, to the production of hydrogen peroxide and other toxic metabolites of dopamine ^44^^,^^45^. Hydrogen peroxide in the presence of Fe2+ and Cu2+ produces hydroxyl radical capable of oxidizing almost any cell structure, which leads to neurodegeneration and contributes to the development of pathologies ^46^. Antioxidants, free radical scavengers, and similar drugs have been potentially used in therapeutic development to prevent Parkinson’s disease. The results of some authors suggest that coumarins and flavonoids can directly capture the reactive oxygen species ^47^. FCS005 had a significant protective effect against oxidative stress, probably because MAO-B inhibitors can reduce oxidative stress by reducing H2O2 production, thus acting as neuroprotective agents ^34^.

FCS005 (3-methyl-7H-furo[3,2-g]-chromen-7-one) is a furocoumarin with a structure similar to that of psoralen, which is isolated from the plant *Psoralea corylifolia* L. and is used traditionally to treat ageing. Psoralen was demonstrated to exhibit *in vitro* inhibitory actions on MAO activities in rat brain mitochondria, preferentially inhibiting MAO-A activity over MAO-B activity ^48^ while FCS005 exhibited selective MAO-B inhibitory activity, which could be attributed to the substitution at position 3; this is the only difference between the two structures.

In conclusion, the coumarin analogue FCS005 synthesized in this research led to the reversal of hypokinesia in the reserpine and levodopa models of Parkinson’s disease and demonstrated anticataleptic effects. Besides, it showed selective inhibitory activity towards MAO-B, as well as antioxidant activity. These results attribute interesting properties to FCS005 supporting further investigations on its potential as an antiparkinsonian agent including the use of models useful to asses not only the control of symptoms but, especially, neuroprotection.
